# Templated Synthesis of Diamond Nanopillar Arrays Using Porous Anodic Aluminium Oxide (AAO) Membranes

**DOI:** 10.3390/nano13050888

**Published:** 2023-02-27

**Authors:** Chenghao Zhang, Zhichao Liu, Chun Li, Jian Cao, Josephus G. Buijnsters

**Affiliations:** 1 State Key Laboratory of Advanced Welding and Joining, Harbin Institute of Technology, Harbin 150001, China; 2 Department of Precision and Microsystems Engineering, Faculty of Mechanical, Maritime and Materials Engineering, Delft University of Technology, Mekelweg 2, 2628 CD Delft, The Netherlands

**Keywords:** template-assisted growth, diamond nanopillars, anodic aluminium oxide (AAO), chemical vapor deposition (CVD), stress modeling

## Abstract

Diamond nanostructures are mostly produced from bulk diamond (single- or polycrystalline) by using time-consuming and/or costly subtractive manufacturing methods. In this study, we report the bottom-up synthesis of ordered diamond nanopillar arrays by using porous anodic aluminium oxide (AAO). Commercial ultrathin AAO membranes were adopted as the growth template in a straightforward, three-step fabrication process involving chemical vapor deposition (CVD) and the transfer and removal of the alumina foils. Two types of AAO membranes with distinct nominal pore size were employed and transferred onto the nucleation side of CVD diamond sheets. Subsequently, diamond nanopillars were grown directly on these sheets. After removal of the AAO template by chemical etching, ordered arrays of submicron and nanoscale diamond pillars with ~325 nm and ~85 nm diameters were successfully released.

## 1. Introduction

Diamond has promising applications in a number of areas such as biomedical engineering [1], photonics [2], electrochemistry [3], and the cutting tools industry [4] owing to its high hardness, high Young’s modulus, high thermal conductivity [5], tunable electrical conductivity [6], and bioinertness/biocompatibility [7], among others. The manufacture of miniature functional diamond structures, including diamond nanowires [8], nanopillars [9], micropillars [10], and pyramids [11] and arrays thereof, has attracted significant attention in the fields of nanoscale quantum sensing, photonic systems, scanning probes, and electrochemical sensors [12]. Currently, numerous methods using top-down or bottom-up synthetic strategies are being used in diamond micro- and nanofabrication [13,14,15,16].

Top-down fabrication methods are the most widely applied, particularly the photolithography process coupled with reactive ion etching (RIE) [17]. Diamond single crystals or polycrystalline diamond films synthesized by chemical vapor deposition (CVD) can be etched anisotropically to produce periodic submicroscale cylinder arrays or diamond nanowires by using, among others, metal droplets [18,19], silica microspheres [20], or nanodiamond particles [21] as masks. The fabrication of periodic high-aspect-ratio nanopillar arrays by e-beam lithography (EBL) and the inductively coupled plasma (ICP) etching method has been demonstrated as well [22,23]. Maskless RIE techniques have been developed to generate diamond nanocone-patterned surfaces [24] and diamond nanowires from heavily boron-doped diamond (BDD) films [25]. Additionally, laser-induced periodic surface structuring (LIPSS) has been reported as a cost-effective technique to nanostructure the surface of BDD for electrode applications [26].

On the other hand, the bottom-up fabrication approach provides excellent opportunities for the rapid and precise manufacture of diamond micro and nano structures but has been explored less extensively. In general, template-assisted CVD synthesis is a popular method for the formation of 1D nanostructures [27]. Regarding the synthesis of diamond nanostructures, various template materials have been explored successfully so far, including carbon nanotubes, black silicon, and anodic aluminium oxide (AAO). The formation of diamond nanorods via a post-synthesis conversion of multiwalled carbon nanotubes in a hydrogen plasma [28,29] results in core-shell structures of diamond (inner core 4–8 nm in diameter) and amorphous carbon (outer shell), which is unfavorable for many applications. Alternatively, the use of nanostructured silicon as the growth template in diamond CVD enables the fabrication of Si/diamond nanorod forests [30] which find application in electrochemistry and as antibacterial surfaces.

AAO is a porous material typically with a hexagonal arrangement of cylindrical pores that provide an ideal template for the preparation of a diverse range of nanostructures, such as nanodots, nanowires, and nanotubes [31,32]. Key advantages of using AAO are the high ordering and close-packed distribution of pores within the template, the controllable structural features (pore diameter [33], pore density, template thickness, etc.), and the relatively simple and inexpensive fabrication of porous AAO membranes via electrochemical anodization [31]. The target materials are commonly deposited within the nanopores via electrochemistry or wetting infiltration, followed by the removing of the sacrificial AAO templates. Ultrathin AAO membranes with a low ratio of thickness and pore diameter have been successfully developed as masks in RIE-based top-down fabrication [34] and in multiple CVD and physical vapor deposition (PVD) processes [35,36]. Wang et al. demonstrated the top-down fabrication of hierarchical nanoporous patterns in diamond by using an RIE with an ultrathin alumina membrane as a mask [37]. To date, however, only very few reports exist on the application of sacrificial AAO in the template-assisted growth of diamond nanostructures. Diamond nanocylinders with differently shaped cross-sections (i.e., circular, square, and triangular) could be synthesized via microwave plasma-assisted CVD with the aid of 50 nm sized diamond particle seeds positioned at the bottom of the AAO membranes [38,39]. However, the coating of diamond nanoparticles at only the bottom side of the thin AAO sheets is complex and not easy to reproduce. Consequently, the controllable and reproducible synthesis of diamond nanowires using AAO still remains a challenge. Recently, Sartori et al. [40] demonstrated the growth of diamond micropillar arrays by placing a porous silicon template onto a continuous CVD diamond thin film, presenting a potential new strategy for AAO-assisted growth of diamond nanostructures as such.

In this work, we report a novel series of experimental steps towards the fabrication of diamond nanopillar arrays by using porous AAO templates. Ultrathin AAO membranes and the nucleation side [41,42] of pre-grown CVD diamond sheets were used as the template and the target substrate, respectively. Nucleation of the diamond nanopillars can proceed directly on the target substrate, thereby eliminating a seeding step involving diamond nanoparticles. The morphology and phase composition of the nanocrystalline diamond pillars were characterized before and after removal of the AAO templates, and the confinement relationship and physical interaction between the AAO membranes and diamond nanopillars are briefly discussed.

## 2. Materials and Methods

### 2.1. Materials

Ultra-thin alumina membranes with hexagonally ordered circular nanopores of average pore size of ~300 and ~70 nm and complementary thickness of 1.6 and 0.2 μm, respectively, were purchased from Shenzhen Topmembranes Technology Company (Shenzhen, China). Potassium hydroxide (KOH), acetone (C_3_H_6_O), and phosphoric acid (H_3_PO_4_) were purchased from Sigma Aldrich (Zwijndrecht, The Netherlands). Demi water (~18 MΩ, ELGA Purelab UHQ) was used to prepare the aqueous solutions. All the reagents were used without further purification.

### 2.2. Diamond Nanopillar Growth

**Figure 1:**
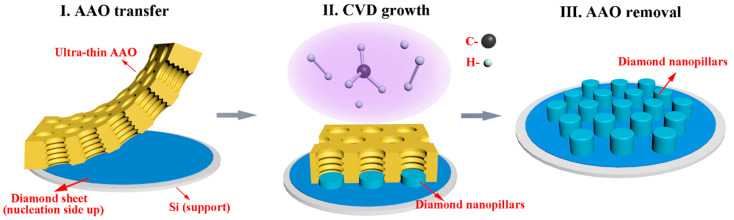
Schematic diagram of the fabricating process of diamond nanopillar arrays by using AAO templates.

Small pieces (10 × 10 mm^2^) cut from a silicon wafer coated with a polycrystalline boron-doped diamond film (4 μm thickness) [26] were treated in a boiling KOH aqueous solution (10 M) for 3 h until the silicon substrate had been removed completely. The released diamond sheets were carefully cleaned multiple times in demi water, transferred onto a fresh piece of a silicon support (10 × 10 mm^2^), and then used as the target substrate in the template-assisted synthesis of diamond nanopillar arrays. Figure 1 illustrates the fabrication steps of the diamond nanopillar growth. In a first step, the ultra-thin AAO membranes (5 × 5 mm^2^) were transferred onto the smooth nucleation side of the diamond sheets in liquid environment of acetone and supported by an additional silicon piece. Following this, the sample stacks were loaded into an in-house-built hot-filament chemical vapor deposition (HFCVD) chamber for diamond growth. During the deposition process, the temperature of the substrate was set at ~725 °C and the flow rates of the reactive gases CH_4_ and H_2_ were 6 and 300 SCCM, respectively. The gas pressure was kept at 10 mbar and a total growth time of 0.5 h or 3 h was chosen for the use of AAO templates with nominal pore sizes of either 70 nm or 300 nm, respectively. Subsequently, to release the diamond nanopillars, the AAO templates were chemically removed by immersion in concentrated H_3_PO_4_ at 200 °C for 2 h. Finally, the nanostructured all-diamond samples were cleaned with demi water. In addition, a diamond thin film was grown on a flat silicon substrate using identical HFCVD conditions but without the use of any AAO template.

### 2.3. Structure and Morphology Characterization

The morphology of the AAO templates and diamond nanopillar arrays was examined by using scanning electron microscopy (SEM, JEOL JSM-6010LA and JSM-6500F). The size distributions of the AAO nanopores and diamond pillars were derived from SEM images by using ImageJ software (version 1.53c). Atomic force microscopy (AFM, Nanosurf Nanite B) was used to examine the surface morphology of the diamond membrane used as target substrate and of the diamond film grown without AAO template. Raman spectroscopy measurements (spot size < 1 μm) were performed with a Horiba LabRAM HR setup, equipped with an argon ion laser operating at 514 nm and spectral resolution of ~0.3 cm^−1^ to study the bonding structure and stress state of the flat diamond thin film and the diamond nanopillars before and after removal of the AAO templates.

### 2.4. Stress Modeling

Residual stress distribution of as-grown diamond pillars, i.e., with the two-dimensional confinement by the AAO skeleton, was modeled by using the finite element method in the software of Abaqus 6.13. The model representing the hexagonal pillar arrays was built in Abaqus CAE and periodic boundary conditions were applied [43]. The model consists of both the diamond part and AAO skeleton part. A diamond cylinder with 325 nm diameter was located at the center, and four one-quarter diamond cylinders were positioned at the corners of the unit cell. The center distance of two diamond cylinders was set to 450 nm. An AAO skeleton with 1.6 μm thickness was created to cover each diamond pillar. The height of the diamond pillars was set at 650 nm, while a diamond layer with a thickness of 4 μm was bonded to the bottom of the pillar arrays. The diamond pillars were initially embedded within the AAO nanopores and both the diamond and AAO were free to expand and shrink. The inner wall surfaces of the AAO nanopores were bonded with the cylinder surfaces of the diamond pillars. To realize the periodic boundary conditions along the x and y axis, each node of the x-z and y-z planes in this model was constrained with the node in the symmetrical position. The whole model was firstly set at 725° C and then cooled down to 20 °C.

## 3. Results and Discussion

### 3.1. Growth of Diamond Pillars with ~300 nm Diameter

**Figure 2:**
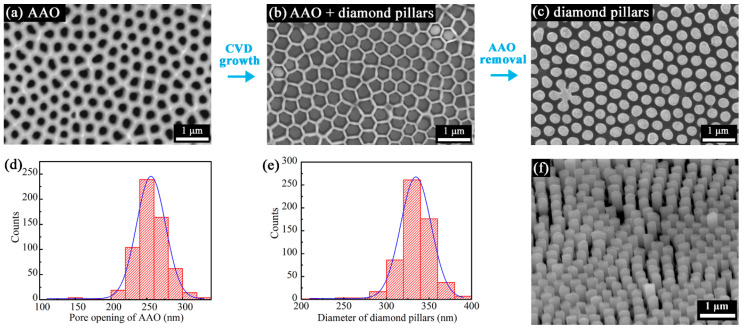
Characterization of AAO membrane and as-grown diamond pillars with nominal diameter of ~300 nm. Top-view SEM images of (**a**) AAO membrane, (**b**) diamond pillars within AAO membrane, and (**c**) released diamond pillars without AAO (note: images not from same location). Frequency distribution histograms of (**d**) AAO nanopores and (**e**) diamond nanopillars as derived from multiple SEM images. (**f**) SEM image of tilted surface (approximately 30°) of released diamond pillars.

The AAO membrane with aspect ratio of ~5 (i.e., thickness of 1.6 μm to nominal pore size of ~300 nm) was used for the synthesis of diamond submicron pillars. The flexible membrane was transferred onto the nucleation side of the polycrystalline diamond sheet (step I in Figure 1). The main reason for using the nucleation side as the target substrate is its extreme smoothness, as shown in the AFM image of Figure S1. The low arithmetic mean surface roughness (S_a_) of ~0.8 nm is beneficial for the adhesion of the AAO membrane via van der Waals interactions. The Raman spectrum recorded from the diamond sheet is shown in Figure S2a, which clearly shows a heavily boron-doped diamond (BDD) composition [44]. An SEM micrograph of the porous AAO membrane used as the growth template is shown in Figure 2a. It features a porous structure with relatively broad distribution of surface pore openings and inter-pore distances. The somewhat disordered pore architecture results from the nearest neighbors being not necessarily all arranged hexagonally as determined by the radial distribution function [33]. Figure 2d presents a statistical distribution of the AAO nanopore openings, which are mainly distributed from 200 nm to 325 nm. A representative lower magnification SEM micrograph of the AAO template and binary representation thereof used for obtaining statistical distribution data are displayed in Figure S3.

After CVD growth for 3 h (step II in Figure 1), the sample was cleaned for 10 min in hot H_3_PO_4_ acid to make it easier for SEM imaging and to better reveal the hexagonal order of the nanopores, as shown in Figure 2b. Close observation of the SEM image shows that well-arranged diamond pillars were grown vertically within each of the AAO nanopores. Figure 2c,f present top-view and tilted-view images of the released diamond pillars after the AAO membrane was completely removed by H_3_PO_4_ acid (step III in Figure 1). It was estimated that the average height of the pillars is around 650 nm, while very few pillars extend to ~1 μm. Clearly, diamond was grown selectively as pillars only within the AAO pores by 2D confinement during the CVD process. Variation in pillar morphology (i.e., diameter and height) appears to be related to the specific shape of the nanopores wherein they were formed. In particular, the flower-shaped diamond pillar cluster near left lower corner of Figure 2c originates from pore defects of the AAO membrane. Neighboring nanopores were most likely interconnected and formed a branched local network of pores accessible to the diamond growth precursors during CVD. Figure 2e shows the statistical distribution of the diamond pillar diameters, which range mainly from 275 nm to 375 nm. These values are significantly larger than the AAO nanopore openings. This may be attributed to the fact that the SEM contrast of the AAO pore walls is less pronounced than that of the submicron diamond pillars, which results in a slight underestimation of the pore openings. Additionally, it should be noted that the ultrathin AAO has trumpet-shaped nanopores with a hexagonal pore opening and a round-shaped AAO pore interior [45,46]. The diamond is mostly grown in the round-shape part of the AAO pores, which may explain the difference in shape between pore openings (hexagonal) and pillars (round). In addition, the AAO template underwent a phase transition [47] and volume change [48] during the heating cycle of the CVD process, which widened the AAO nanopores during the diamond growth. 

**Figure 3:**
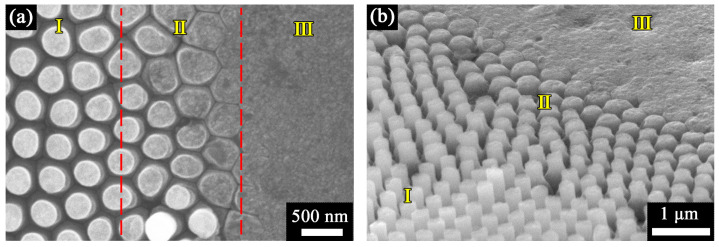
SEM images of diamond nanopillars at the boundary region: (**a**) top view and (**b**) tilted view. Regions I, II, and III indicate the distinct zones of templated growth of pillar array, transition zone, and plain growth of diamond, respectively.

Similar to previous work on the synthesis of diamond micropillar arrays by using porous silicon membranes [40], three distinct growth zones were distinguished at the periphery of the contact area formed with the AAO template (Figure 3). Well-arranged diamond pillars of similar height and with a flat top surfaces were grown in the main region with zero gap between the diamond sheet and AAO template. Submicron diamond pillars of decreasing height and with increasing roundness of the top surface were formed in the transition area (~2 μm wide), while in the outer region, the gap was too large and the diamond was grown flat as a nanocrystalline diamond (NCD) layer without any nanostructuration.

### 3.2. Growth of Diamond Nanopillars with ~70 nm Diameter

**Figure 4:**
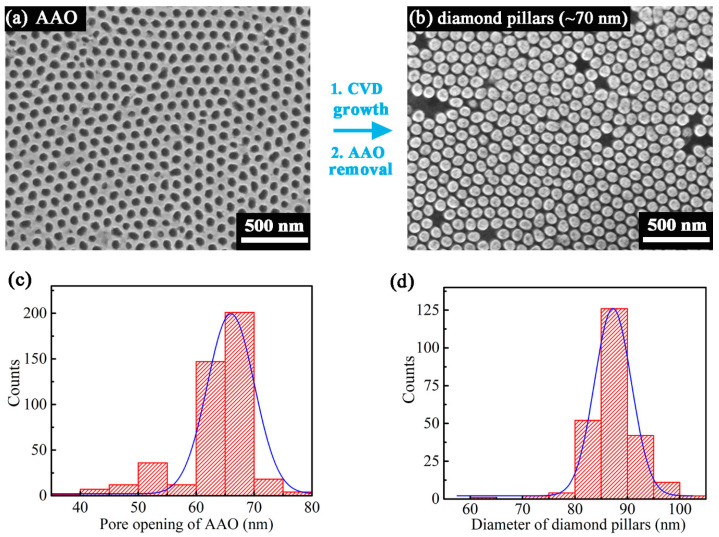
Characterization of AAO membrane and as-grown diamond pillars with nominal diameter of ~70 nm. SEM images of (**a**) AAO membrane and (**b**) released diamond pillars. Frequency distribution histograms of (**c**) AAO nanopores and (**d**) diamond nanopillars.

To extend the scope of fabricating diamond pillars by the proposed template-assisted method, the other AAO template type with nominal pore size of ~70 nm was used for growing diamond nanopillars. The AAO membrane with thickness of 200 nm and aspect ratio of ~3 exhibits a porous structure with perceivably round shaped surface pore openings (Figure 4a). Overall, the degree of hexagonal ordering of pores is high but the membrane is composed of many small regions (ranging from about 350 to 750 nm in size) in which the nanopores are in ordered array. At the boundary of neighboring regions [49], nanopores of reduced ordering and distinct pore size (typically smaller) are found. The respective statistical distribution of AAO pore openings is displayed in Figure 4c.

After AAO transfer, CVD growth, and AAO removal, arrays of well-arranged diamond nanopillars with ~85 nm diameter and ~200 nm height were obtained, as shown in Figure 4b. The statistical distribution of as-obtained diamond nanopillars is displayed in Figure 4d. Again, the overall diameter of grown pillars is somewhat larger than that of the AAO pore openings. Few surface spots did not feature any diamond nanopillars, most likely due to an incomplete through-membrane porosity or a too narrow pore opening/diameter for the CVD growth precursors to diffuse through.

Obtained results demonstrate the strong viability of presented approach in the manufacturing of nanostructured diamond, even for features smaller than 100 nm. The scale of pattern transfer is limited to mm size range because of the finite contact area between template and substrate but wafer bonding could be explored in follow-up research to achieve AAO-on-silicon [50] or AAO-on-diamond. As-produced diamond nanopillar arrays may find extensive application in areas where well-arranged diamond nanostructures are wanted, such as skeletal tissue engineering [51], optics [52], and electrochemical sensing [53]. 

### 3.3. Raman Spectra and Residual Stress

**Figure 5:**
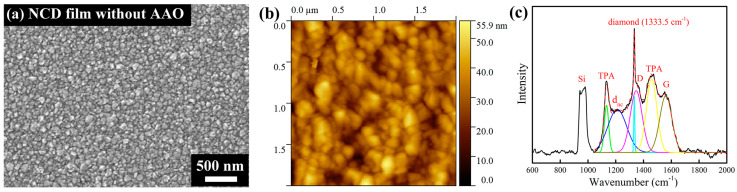
Characterization of the nanocrystalline diamond film grown on a flat Si wafer without AAO membrane. (**a**) SEM image, (**b**) AFM topographic image (2 × 2 μm^2^) and (**c**) Raman spectrum with peak deconvolution.

For comparison, a non-patterned diamond film was grown under identical HFCVD conditions on a flat Si wafer without any AAO template. The SEM and AFM images of the film are shown in Figure 5a,b, respectively. A dense and highly uniform NCD layer was obtained with a maximum grain size of about 100 nm. The Raman spectrum and its deconvolution results according to [54] are displayed in Figure 5c. A sharp and intense diamond peak is located at 1333.5 cm^−1^, demonstrating good diamond quality. In addition, Raman signals originating from the Si substrate (935–990 cm^−1^) and from various carbon constituents present in the grain boundaries can be observed as well, namely the D-band (around 1355 cm^−1^) and G-band (around 1580 cm^−1^) originating from sp^2^ hybridized carbon present in both disordered/defective and crystalline forms of graphite material and trans-polyacetylene (peaks near 1140 cm^−1^ and 1470 cm^−1^ denoted as TPA). The band near 1200 cm^−1^ (labeled as d_nc_) is attributed to the broadened vibrational density of states of small diamond clusters and/or tetrahedral amorphous carbon.

**Figure 6:**
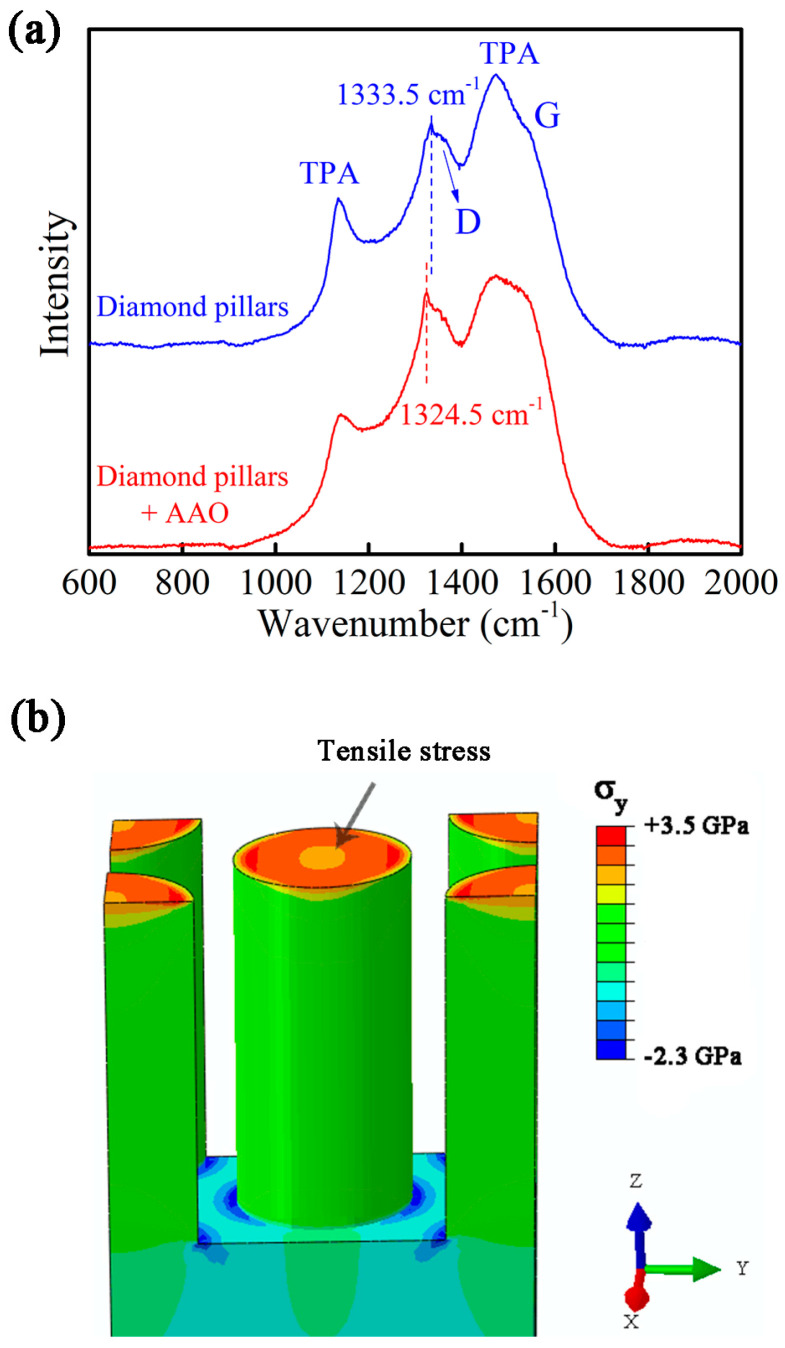
(**a**) Raman spectra of the diamond pillars (~325 nm in diameter) before and after removal of the AAO template. (**b**) Stress modeling data showing the contour plot of the residual stress along the y-axis in the diamond pillars after the cooling down and before AAO removal. For reasons of clarity, the AAO skeleton is not shown.

Figure 6a shows the Raman spectra of the submicron diamond pillars before (red) and after (blue) removal of the AAO template (see Figure 2). The diamond peak parameters evaluated from the Raman spectra, i.e., peak position and full width at half maximum (FWHM), are listed in Table 1. For the fully released diamond pillars, the Raman peak at 1333.5 cm^−1^ is the typical diamond one-phonon line corresponding to sp^3^ carbon. The D-band (around 1350 cm^−1^), G-band (around 1550 cm^−1^), and the peaks related to trans-polyacetylene (at 1137 cm^−1^ and 1474 cm^−1^) [55] are relatively intense. Note that the Raman spectrum differs significantly from that of the diamond sheet (i.e., deliberately chosen to be heavily doped BDD) used as substrate (Figure S2a) and confirms that the material composition of the pillars is nanocrystalline diamond (NCD). It also differs from that of the NCD layer grown without AAO coverage, which indicates that the confined growth had some effect on the diamond quality. The observed difference is likely due to the change in local growth environment around the membrane pores, as was previously reported for the template-assisted growth of NCD micropillar arrays using commercial porous Si membranes [40]. On the other hand, a similar Raman spectrum was recorded from the AAO-confined diamond pillars. However, the diamond peak displayed a red-shift (1324.5 cm^−1^) compared to the stress-free diamond pillars after AAO removal (1333.5 cm^−1^). This points out the presence of residual stress between AAO skeleton and as-grown diamond pillars. At the end of the CVD process, diamond pillars had been grown that were fully embedded within the AAO nanopores. During the final cooling down step, both the diamond pillars and AAO skeleton shrank at different rates, and residual stress was built up by the mismatch of the coefficient of thermal expansion (CTE) between AAO and diamond. This residual stress was released after the AAO templates were chemically removed by the acid. The relationship between stress and diamond Raman peak shift can be described by Equation (1) [56]

w(P) = w_0_ + α_1_P + α_2_P^2^ (1)

where w is the position of the diamond Raman peak (in cm^−1^), P is the value of stress (in GPa), w_0_ = 1333.0 cm^−1^, α_1_ = 2.83 cm^−1^ GPa^−1^, and α_2_ = −3.65 × 10^−3^ cm^−1^ GPa^−1^. Considering the value of Raman shift of −9 cm^−1^, a tensile residual stress of 3.2 GPa was calculated. Since the Raman spectral resolution is ~0.3 cm^−1^, the error on the stress estimation is approximately 0.1 GPa.

The Raman spectrum recorded from the as-grown, AAO-confined diamond nanopillars with ~85 nm diameter is given in Figure S2b. Here, the diamond one-phonon line is also red-shifted (1329.5 cm^−1^, see Table 1) compared with the stress-free diamond Raman peak. The TPA peak and G band around 1480 and 1580 cm^−1^, respectively, can be assigned to sp^2^ carbon. Note that the dominant Raman signals between 1050 and 1250 cm^−1^ originate from the BDD sheet used as substrate because the Raman confocal depth (estimated few microns) extends far beyond the thin AAO template of limited thickness (~200 nm).

To further investigate the stress buildup in the AAO-confined diamond pillars, stress modeling was performed. As a first approximation, only the thermal stresses were considered because other origins of stress including intrinsic stress by microstructural evolution (i.e., grain growth) are considered of lesser magnitude. The unit cell shown in Figure S4 was generated as the model to represent the hexagonal diamond pillar arrays. The mechanical properties of the AAO skeleton and CVD diamond used in the modeling are listed in Table S1 and Table S2, respectively.

The modeling results of thermal stress distribution in the diamond pillars are shown in Figure 6b. In particular, the σ_y_ stress is plotted which is a measure of the residual stress along the y-axis. The AAO part is hidden for clarity. Tensile stress is mainly distributed at the top surfaces of the diamond cylinders. From the outer to inner ring, the value of stress decreases gradually from 3.5 GPa to 2.5 GPa. Along the central line of the diamond pillars, the value of tensile stress decreases gradually from top to bottom. Compressive stress is mainly generated within the diamond layer at the proximity of the pillar bottoms. Figure S5 displays the residual stress of the diamond pillars along the x-axis, logically showing a similar result. The calculation results indicate that the tensile stress dominates the top part of the diamond pillars, which is in line with the recorded Raman shift.

The bottom-up approach demonstrated here provides a low-cost, versatile, and applicable method to produce diamond nanopillars with high ordering and controlled geometry. Such nanopillar arrays may find wide application in field emission, thermal management, and ultrasensitive force microscopy and photonic bandgap crystals [38]. Since diamond shows excellent chemical stability and biocompatibility, as-produced diamond pillar arrays are also applicable as the substrate for cell culture. Further, boron doping of such diamond submicron/nanopillars requires only a minor adaptation of the methodology (i.e., adding a boron precursor to the CVD gas mixture) and may support the development of microelectrode arrays (MEAs) for cell signal reception and retinal implants, with tunable electrode shapes and sizes within reach by using different AAO templates.

## 4. Conclusions

In this work, diamond nanopillar arrays were obtained through chemical vapor deposition using AAO membranes with distinct pore size as the growth template. Vertically aligned diamond nanopillars with ~85 and ~325 nm diameter and aspect ratio of ~2.5 were successfully synthesized. The as-grown diamond pillars showed significant stress buildup and stress release, respectively, before and after AAO removal. From stress modeling, it is concluded that this is linked to the mismatch in thermal expansion coefficient between the AAO and nanocrystalline diamond components. The AAO-assisted fabrication of diamond nanopillar arrays may find wide application in skeletal tissue engineering, optics, and electrochemical sensing. Depending on the application scenario, optimization of the nanopillar characteristics (e.g., diamond grain size, pillar aspect ratio, material doping) may be required. The demonstrated bottom-up synthesis approach could also be further exploited in future studies to reach large-area pattern transfer and to prepare diamond pillars with different morphology by using AAO templates with triangular, square, or even multi-level hierarchical pore architectures.

## Figures and Tables

**Table 1:** Diamond peak parameters evaluated from the Raman spectra of the various NCD samples.

Sample	Diamond Peak Position (cm^−1^)	FWHM of Diamond Peak (cm^−1^)
Flat NCD film	1333.5 ± 0.3	8.6
NCD pillars (~325 nm) before AAO removal	1324.5 ± 0.3	16.2
NCD pillars (~325 nm) after AAO removal	1333.5 ± 0.3	15.8
NCD pillars (~85 nm) before AAO removal	1329.5 ± 0.3	19.2

## Data Availability

The data from this study are available upon request.

## Supplementary Materials

The following supporting information can be downloaded at: https://www.mdpi.com/article/10.3390/nano13050888/s1, Figure S1: AFM topographic image (10 × 10 μm^2^) of the nucleation side of the diamond sheets; Figure S2: Raman spectra recorded from (a) nucleation side of the diamond sheet and (b) as-grown diamond nanopillars with ~70 nm diameter before removal of the AAO template; Figure S3: Conversion of SEM images (left column) into binary representations (right column) of AAO template (a,b) and diamond pillars (c,d); Figure S4: Finite element model of the diamond pillar array with the unit cell indicated in red; Figure S5: Stress modeling data showing the contour plot of the residual stress along the x-axis in the diamond pillars after the cooling down and before AAO removal. For reasons of clarity, the AAO skeleton is not shown; Table S1: Material performance parameters of AAO skeleton in FEM; Table S2: Material performance parameters of diamond in FEM. References [57,58,59,60,61] are cited in the supplementary materials.
