# Supplementary material for: Templated Synthesis of Diamond Nanopillar Arrays Using Porous Anodic Aluminium Oxide (AAO) Membranes
Source: Nanomaterials (Basel). 2023 Feb 27;13(5):888. doi: 10.3390/nano13050888 (PMC10004781; doi:10.3390/nano13050888)
Supplement: Supplementary file 1 [file nanomaterials-13-00888-s001.zip › nanomaterials-2228108-supplementary.pdf]

Supporting Information for

Templated synthesis of diamond nanopillar arrays using porous anodic aluminium oxide (AAO) membranes

Chenghao Zhang [1, 2], Zhichao Liu [2], Chun Li [1], Jian Cao [1] and Josephus G. Buijnsters [2,*]

[1] State Key Laboratory of Advanced Welding and Joining, Harbin Institute of Technology, Harbin 150001, China

[2] Department of Precision and Microsystems Engineering, Research Group of Micro and Nano Engineering, Delft University of Technology, Mekelweg 2, 2628 CD Delft, the Netherlands

* Correspondence: j.g.buijnsters@tudelft.nl (J.G.B.)

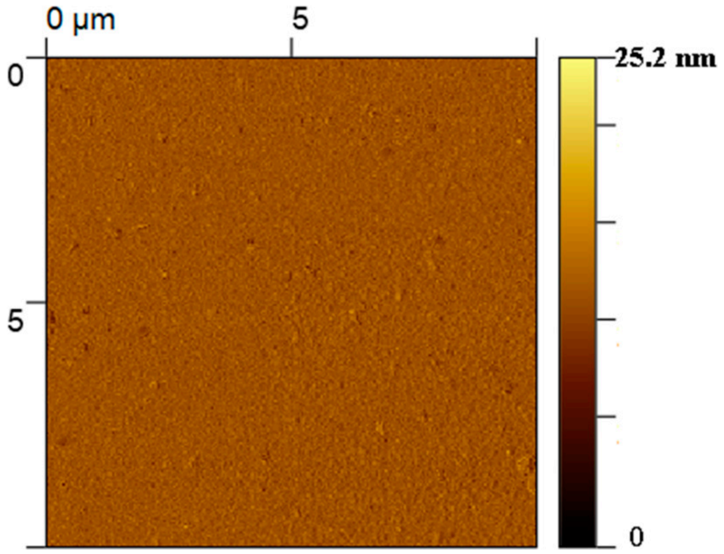

Figure S1. AFM topographic image (10×10 µm^2) of the nucleation side of the diamond sheets.

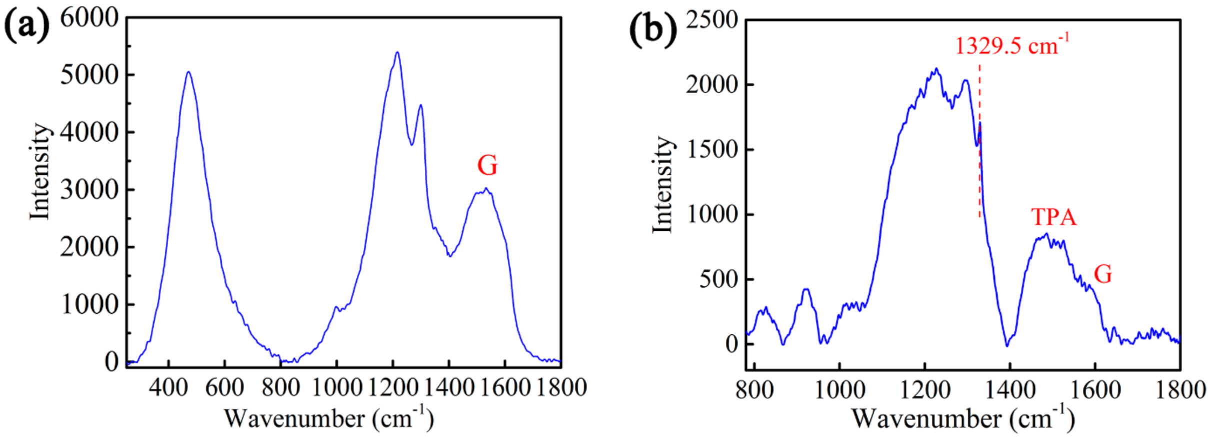

Figure S2. Raman spectra recorded from (a) nucleation side of the diamond sheet and (b) as-grown diamond nanopillars with ~70 nm diameter before removal of the AAO template.

Assignment of Raman peaks

The peaks around 1310 and 1329.5 cm^{-1} are the distorted and shapely diamond one-phonon lines, characteristic of heavily B-doped and undoped diamond, respectively. The bands around 500 cm^{-1} and 1200 cm^{-1} can be assigned to a combination of electronic Raman scattering and a Fano-shaped band, and the maximum of phonon density of states, respectively. The G band at 1580 cm^{-1} originates from sp^2 carbon in the grain boundaries and the signal at about 1480 cm^{-1} is related to trans-polyacetylene (TPA).

Note that the diamond sheet (Figure S2a) and grown diamond pillars (Figure S2b) were deliberately chosen to be of different composition, namely heavily boron-doped diamond (BDD) and undoped nanocrystalline diamond, respectively. As a consequence, the Raman spectral features clearly marked the distinction between both diamond material types, even for such tiny pillars.

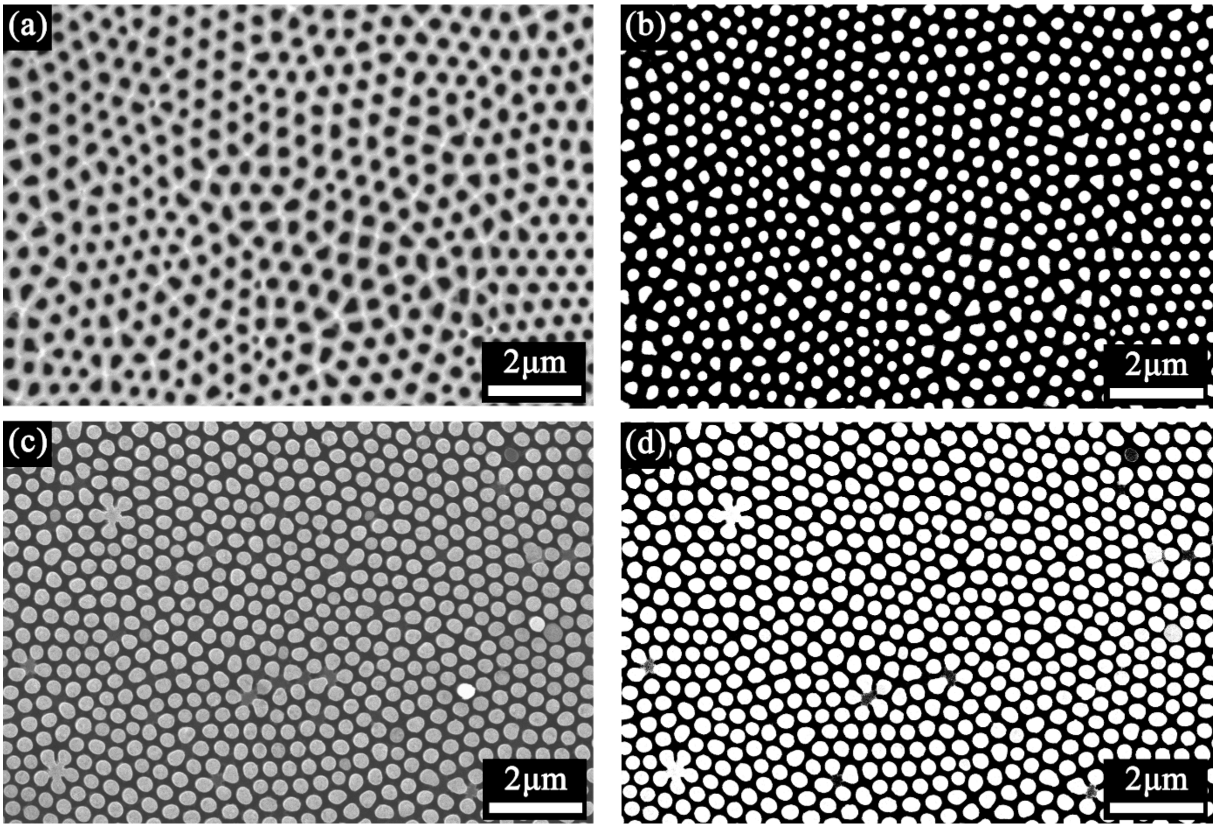

Figure S3. Conversion of SEM images (left column) into binary representations (right column) of AAO template (a,b) and diamond pillars (c,d).

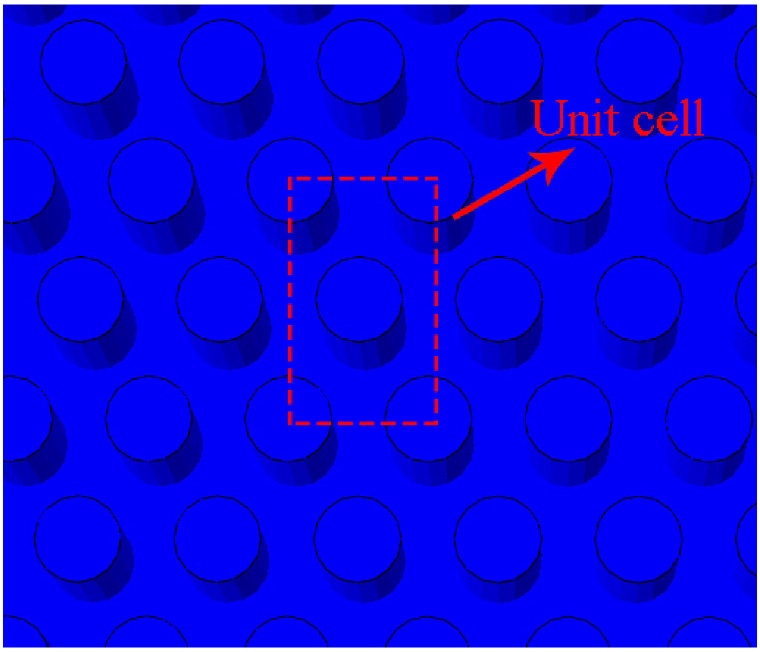

Figure S4. Finite element model of the diamond pillar array with the unit cell indicated in red.

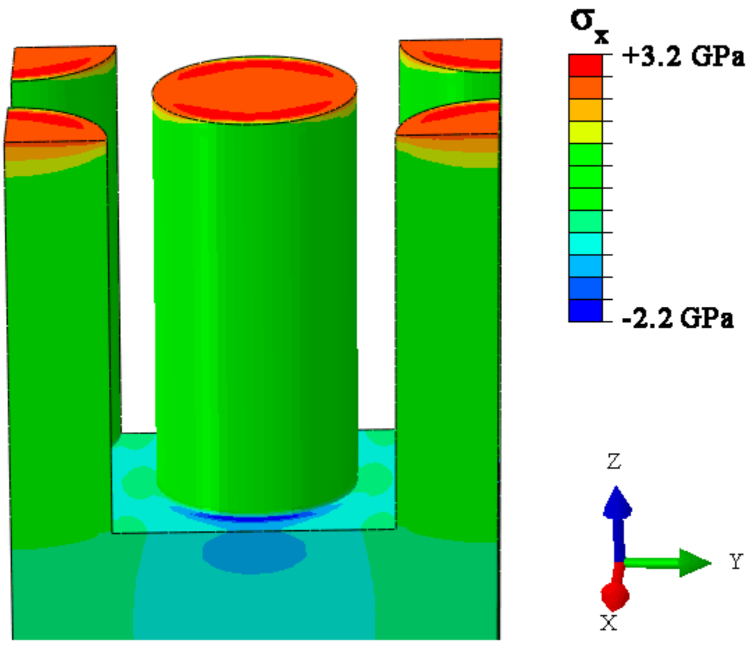

Figure S5. Stress modelling data showing the contour plot of the residual stress along the x-axis in the diamond pillars after the cooling down and before AAO removal. For reasons of clarity, the AAO skeleton is not shown.

Table S1. Material performance parameters of AAO skeleton in FEM.

Temperature (°C)	Young's modulus (GPa) [57]	Poisson's ratio [57]	Temperature (°C)	Thermal expansion coefficient (10^{-6} K^{-1}) [58]
20-900	253	0.24	100	12.401
			200	12.592
			300	13.110
			400	13.009
			500	12.950
			600	12.690
			700	12.507
			800	12.042

Table S2. Material performance parameters of diamond in FEM.

Temperature (°C)	Young's modulus (GPa) [59]	Poisson's ratio [60]	Temperature (°C)	Thermal expansion coefficient (10^{-6} K^{-1}) [61]
20	895	0.034	207	1.701
400	857.77	0.034	307	2.042
800	820.54	0.034	407	2.333
			507	2.598
			607	2.819
			707	3.019
			807	3.219

References

[57] Gallas, M.R.; Piermarini, G.J. Bulk modulus and Young's modulus of nanocrystalline γ-alumina. *J. Am. Ceram. Soc.* **1994**, *77*, 2917–2920. https://doi.org/10.1111/j.1151-2916.1994.tb04524.x.

[58] Balakrishnan, G.; Thirumurugesan, R.; Mohandas, E.; Sastikumar, D.; Kuppusami, P.; Song, J.I. Phase transition and thermal expansion studies of alumina thin films prepared by reactive pulsed laser deposition. *J. Nanosci. Nanotechno.* **2014**, *14*, 7728–7733. https://doi.org/10.1166/jnn.2014.9480.

[59] Seing, Y.; Nagai, S. Temperature dependence of the Young's modulus of diamond thin film prepared by microwave plasma chemical vapour deposition. *J. Mater. Sci. Lett.* **1993**, *12*, 324–325. https://doi.org/10.1007/bf01910092.

[60] Mohr, M.; Caron, A.; Herbeck-Engel, P.; Bennewitz, R.; Gluche, P.; Brühne, K.; Fecht, H.J. Young's modulus, fracture strength, and Poisson's ratio of nanocrystalline diamond films. *J. Appl, Phys.* **2014**, *116*, 124308. https://doi.org/10.1063/1.4896729.

[61] Pickrell, D.J.; Kline, K.A.; Taylor, R.E. Thermal expansion of polycrystalline diamond produced by chemical vapor deposition. *Appl. Phys. Lett.* **1994**, *64*, 2353–2355. https://doi.org/10.1063/1.111612.
